# Mechanistic insights into the inhibitory effect of epigallocatechin gallate on the production of virulence factors in *Proteus mirabilis*

**DOI:** 10.1097/MD.0000000000046893

**Published:** 2026-01-02

**Authors:** Zhichun Xue, Huiling Chen, Yuying Qi, Qin Zeng, Gang Yan, Guiju Fang

**Affiliations:** a Hospital Infection Management Department, Ningde Municipal Hospital of Ningde Normal University, Ningde, China; b College of Marine Sciences, Ningde Normal University, Ningde, China; c Clinical Laboratory, Ningde Municipal Hospital of Ningde Normal University, Ningde, China; d Department of Respiratory and Critical Care Medicine, Ningde Municipal Hospital of Ningde Normal University, Ningde, China.

**Keywords:** epigallocatechin gallate, inhibiton, *Proteus mirabilis*, virulence factors

## Abstract

This study aimed to elucidate the inhibitory effects of epigallocatechin gallate (EGCG) on virulence factors of *Proteus mirabilis*, a primary agent in catheter-associated urinary tract infections. The antimicrobial efficacy of EGCG was evaluated using the microdilution method, and the presence of biofilms and crystal formation were visualized using Gram staining and microscopy. Biochemical assays were performed to assess the inhibitory effects of EGCG on urease and hemolysin production. Swarming motility, a key determinant of bacterial colonization and spread, was evaluated using specialized swarming assays. Furthermore, the modulation of virulence-related gene expression by EGCG was quantitatively analyzed using quantitative real-time PCR, providing insights into its molecular mechanisms of action. EGCG exhibited a minimum inhibitory concentration of 94 μg/mL against *P mirabilis*, with a minimum bactericidal concentration of 188 μg/mL. EGCG significantly inhibited bacterial growth and hindered biofilm formation, and its inhibitory effect was positively correlated with EGCG concentration. EGCG markedly suppressed urease, hemolysin, and swarming motility, leading to notable reduction in crystal production. Furthermore, EGCG significantly downregulated the expression of virulence genes including *flhB*, *flhD*, *speA*, *luxS*, *ureR*, *hpmA*, and *hpmB*. EGCG demonstrated multifaceted inhibitory effects against *P mirabilis*, including growth, biofilm formation, crucial enzyme production, swarming motility, and inhibition of key virulence genes. These findings establish a robust experimental foundation for utilizing EGCG to treat infections induced by *P mirabilis*.

## 1. Introduction

Catheter-associated urinary tract infections (CAUTIs) account for a significant proportion (25%) of hospital-acquired infections. These infections are a major concern, as they markedly increase patient mortality, healthcare costs, and duration of hospital stay.^[1,2]^ A variety of pathogens are implicated in CAUTIs, including *Escherichia coli*, *Pseudomonas aeruginosa*, *Enterococcus* species, coagulase-negative *Staphylococci*, several non-fermenting bacterial species, and *Candida* species.^[3]^ Among these, *Proteus mirabilis* stands out because of its array of virulence factors, such as urease production, hemolysin, and swarming motility, which play pivotal roles in its pathogenicity.^[4,5]^

Urease mediates the hydrolysis of urea into ammonia and carbon dioxide, consequently increasing the urine pH. This shift promotes the precipitation of calcium and magnesium ions, contributing to urinary stone formation and potential urinary tract obstruction.^[5,6]^ Furthermore, the swarming motility of *P mirabilis* enables retrograde entry into the bladder, initiating infection.^[7]^ This motility is modulated by *flhB* and *flhD* genes through flagellar mediation. Hemolysin plays a crucial role in the ability of bacteria to invade and penetrate cellular barriers by lysing the renal tubular epithelial cells.^[8]^ Additionally, biofilms play a pivotal role in shielding bacteria from host immune responses and antibiotic assaults.^[9]^

Strategically targeting virulence factors in *P mirabilis* is essential for the effective management of CAUTIs. However, increasing antibiotic resistance limits the efficacy of antibiotics, despite their dependency on CAUTIs treatment.^[10,11]^ This challenge has sparked increased interest in exploring natural plant extracts as alternative therapeutic strategies against CAUTIs.^[12]^ Research has shown that compounds such as allicin and quercetin significantly reduce the urease activity, biofilm formation, and quorum sensing capabilities of *P mirabilis*.^[13]^ Moreover, quercetin suppresses the expression of *flhC* and *speB* in a dose-dependent manner, effectively curtailing swarming motility.^[14]^

Epigallocatechin gallate (EGCG), a prominent active component of tea polyphenols and member of the catechin family, is recognized for its broad-spectrum biological activities, including antibacterial, antiviral, antioxidant, anti-atherosclerotic, antithrombotic, antiangiogenic, anti-inflammatory, and antitumor properties.^[15–18]^ Although previous research has examined the effects of EGCG on *P mirabilis*, comprehensive studies are scarce.^[19,20]^ This study evaluated the inhibitory effects of EGCG on critical virulence factors of *P mirabilis*, including urease activity, biofilm development, hemolysin production, and swarming behavior, while also analyzing the expression of related genes using quantitative real-time polymerase chain reaction (qRT-PCR). The outcomes of this study are expected to provide a foundation for leveraging plant extracts in the development of innovative approaches for the prevention and treatment of CAUTIs.

## 2. Materials and methods

### 2.1. Bacterial strain and culture condition

*P mirabilis* strain (ATCC 49055), obtained from the Shanghai Microbial Strain Collection Center and preserved in glycerol at −80°C, was used in this study. For routine culturing, tryptic soy agar, provided by Haibo Biotech (Qingdao, China), was used to ensure consistent experimental conditions. Bacterial suspensions were prepared according to the protocol described by Durgadevi et al,^[21]^ which involved the cultivation of *P mirabilis* in fresh nutrient broth to achieve an optical density (OD) of 0.4 at 600 nm, corresponding to 1 × 10^8^ CFU/mL. An artificial urine medium was prepared following established protocols, using Luria-Bertani (LB) broth medium (pH 7.2 ± 0.2) provided by Haibo Biotech (Qingdao, China), and supplemented with 2% urea, 0.03% magnesium sulfate heptahydrate, and 0.08% calcium chloride dihydrate.^[22]^ EGCG of ≥99% purity, provided by Yuan Ye Biotechnology (Shanghai, China), was refrigerated at 4°C. Various concentrations of EGCG were prepared using LB broth as the diluent.

### 2.2. Minimum inhibitory concentration (MIC) and minimum bactericidal concentration (MBC) assays

MIC and MBC assays for EGCG were conducted using the microdilution method, in accordance with the Clinical and Laboratory Standards Institute guidelines.^[23,24]^ EGCG solutions, in a concentration range of 3000 to 6 μg/mL in 100 μL volumes, were co-incubated with 5 μL of *P mirabilis* suspension at 37°C for 16 hours. The MIC was defined as the lowest concentration in the wells showing no visible bacterial growth. To determine MBC, aliquots with concentrations exceeding the MIC were plated on plate count agar provided by Haibo Biotech (Qingdao, China) and incubated at 37°C for 16 hours. The MBC was defined as the lowest concentration that resulted in no detectable bacterial growth.

### 2.3. Time-killing growth curve assay

To assess the effect of EGCG on *P mirabilis* growth, 20 mL of EGCG solution at concentrations of 24, 47, and 94 μg/mL was mixed with 1 mL of the bacterial suspension. In contrast, the control group received 1 mL of bacterial suspension without EGCG treatment. These mixtures were incubated at 37°C on a shaker, and the OD at 600 nm was measured at 4-hour intervals to construct the growth curves.^[25]^

### 2.4. Urease production assay

The methodology described by Ranjbar-Omid et al was used to assess urease production.^[13]^ EGCG solutions (3 mL) at concentrations of 24, 47, and 94 μg/mL were mixed with 150 μL of *P mirabilis* suspension for each assay condition. Conversely, the control group was treated with the same volume of solvent without EGCG to maintain equivalent conditions, except for the absence of the compound. The mixtures were incubated at 37°C in a shaker for 16 hours. Following incubation, centrifugation was performed at 12,000 rpm for 10 minutes and the supernatant was collected. Phenol red solution (0.02%) provided by Sangon Biotech (Shanghai, China) was added to the supernatant. The OD at 570 nm was measured to evaluate variations in urease production among the bacterial strains subjected to different concentrations of EGCG.

### 2.5. Hemolysin production assay

To assess the effect of EGCG on hemolysin production in *P mirabilis*, cultures treated with EGCG (24, 47, and 94 μg/mL) were mixed with 2% fresh sheep blood. By contrast, the control group was left untreated. After incubation at 37°C for 3 hours, the OD at 590 nm was measured to assess hemolysin production.^[13]^

### 2.6. Biofilm formation assessment

The effect of EGCG on biofilm formation by *P mirabilis* was assessed by mixing 3 mL of EGCG solutions at concentrations of 24, 47, and 94 μg/mL with 150 μL of bacterial suspension. In contrast, the control group received only the bacterial suspension, without EGCG treatment. The mixture was then incubated at 37°C for 16 hours without agitation. Following incubation, glass slides were washed with 1 × phosphate-buffered saline, stained with crystal violet for 5 minutes, and then rinsed. The bound dye was solubilized in 95% ethanol and the absorbance correlating to the biofilm biomass was measured at OD 540 nm. Gram staining was performed to visualize the biofilms on glass slides, and the morphology of the stained biofilms was observed under an optical microscope.^[26]^

### 2.7. Crystal formation assessment

To assess the effect of EGCG on crystal formation, 3 mL of EGCG solution (24, 47, and 94 μg/mL) in artificial urine medium was mixed with 150 μL of bacterial suspension, while the control group remained untreated. Cells were incubated at 37°C for 16 hours without agitation. Subsequently, the glass slides were removed and crystal formation was directly observed and examined using an optical microscope. The culture medium was centrifuged at 12,000 rpm for 10 minutes, and the concentrations of calcium and magnesium ions were determined in the culture medium using an automated biochemical analyzer (Hitachi, Japan).

### 2.8. Swarming motility assay

LB agar plates supplemented with varying concentrations of EGCG (24, 47, and 94 μg/mL) were prepared to assess their effects on *P mirabilis* swarming motility of *P mirabilis*. In contrast, control plates were prepared without EGCG. All the plates were air-dried at 37°C for 24 hours prior to inoculation. A 5 μL aliquot of the bacterial suspension was then inoculated onto each LB agar plate, followed by incubation at 37°C for 16 hours. The diameter of the resulting bacterial colonies was measured to quantify the swarming motility.^[21]^

### 2.9. qRT-PCR analysis

Gene expression was analyzed by incubating 3 mL EGCG (47 μg/mL) with 150 μL *P mirabilis* suspension at 37°C for 16 hours with shaking. By contrast, the control group did not receive any treatment. Subsequently, total RNA was extracted and cDNA was synthesized using RNA extraction and reverse transcription kits provided by Promega (Shanghai, China). qRT-PCR analysis was performed using a SLAN-965 automated PCR analysis system (SANSURE Biotech, China). The *16S rRNA* gene served as a reference for quantifying the relative expression of the target virulence genes (*flhB*, *flhD*, *speA*, *ureR*, *hpmA*, *hpmB*, and *luxS*). Details of the specific primers used for these genes are presented in Table 1. A sub-MIC concentration of 47 μg/mL EGCG was selected for the qRT-PCR assays. This level was chosen to avoid bactericidal effects that might distort gene expression profiles, while still being sufficient to exert measurable inhibitory effects on virulence phenotypes.

**Table 1:** Selected genes and primers used in qRT-PCR assays.

Gene	Forward sequence	Reference
*16S-F*	GAGTTTGATCATGGCTCAG	[40]
*16S-R*	CCCACTGCTGCCTCCCGT	
*ureR-F*	GGATGTAGCAAAAACGCTCT	[40]
*ureR-R*	ATGCGTCACAAAAATAAGCA	
*hpmA-F*	GTTGAGGGGCGTTATCAAGAGTC	[40]
*hpmA-R*	GATAACTGTTTTGCCCTTTTGTGC	
*hpmB-F*	CAGTGGATTAAGCGCAAATG	[37]
*hpmB-R*	CCTTCAATACGTTCAACAAACC	
*flhD-F*	CTTCCGCAATGTTTAGACTG	[40]
*flhD-R*	ATTTGTTGCAAATCATCCAC	
*flhB-F*	TCAGCTAACGCATTCATTG	[21]
*flhB-R*	GCCAGTGTTTCTAGGCTTG	
*speA-F*	CATCTGTGATCCAAGGTGAA	[21]
*speA-R*	GCCGAACGTTTAGAAGTGAT	
*luxS-F*	ACGATGAAAACACCCTCTGG	[21]
*luxS-R*	CGCATGAAACCCGCAAATAAG	

qRT-PCR = quantitative real-time polymerase chain reaction.

### 2.10. Statistical analysis

To minimize potential sources of bias, such as variability in bacterial growth conditions, batch effects, and operator handling, all experiments were performed under standardized protocols using identical culture media and incubation parameters. Negative and untreated controls were included in each assay, and independent replicates were performed in separate runs to ensure reproducibility and reduce random error. Each experiment was independently repeated at least 3 times to ensure reliability for subsequent statistical analysis. Statistical analyses were conducted using SPSS software (version 23). Differences between the treatment groups were evaluated using one-way analysis of variance and *t* tests, with significance set at *P* < .05.

### 2.11. Ethics statement

This study did not involve human participants or animal experiments. Only standard microbial strains obtained from a recognized culture collection (*Proteus mirabilis* ATCC 49055) were used; therefore, approval from an institutional ethics committee or IRB was not required.

## 3. Results

### 3.1. Antimicrobial activity of EGCG on P mirabilis

**Figure 1.**
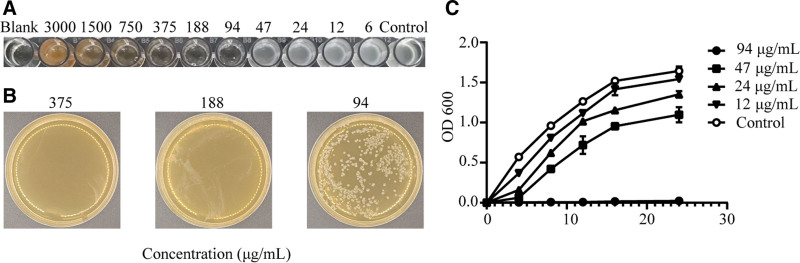
Inhibitory effects of EGCG on *Proteus mirabilis* growth. (A) MIC of EGCG against *P mirabilis*. (B) MBC of EGCG against *P mirabilis*. (C) Growth curves of *P mirabilis* in medium supplemented with varying concentrations of EGCG. Each data point represents the mean ± standard deviation of 3 biological replicates. EGCG = epigallocatechin gallate, MBC = minimum bactericidal concentration.

The antimicrobial efficacy of EGCG against *P mirabilis* was assessed using the microdilution method. The findings revealed a pronounced antimicrobial effect, with an MIC of 94 μg/mL (Fig. 1A) and MBC of 188 μg/mL (Fig. 1B). Notably, the inhibitory effect of EGCG on *P mirabilis* growth increased with increasing concentrations, culminating in complete inhibition of bacterial growth at the MIC value (Fig. 1C).

### 3.2. EGCG’s effect on P mirabilis urease and hemolysin production

**Figure 2.**
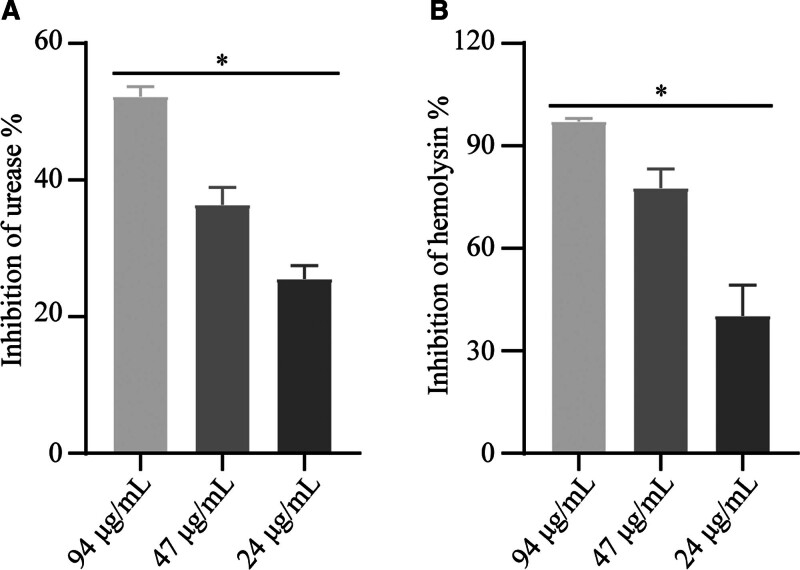
Impact of EGCG on enzyme production in *Proteus mirabilis* cell lysate. (A) Influence of EGCG on urease production. (B) Influence of EGCG on hemolysin production. Data are presented as mean ± standard deviation from 3 biological replicates. Asterisks indicate statistically significant differences between the treated and control groups (*P < .05). EGCG = epigallocatechin gallate.

Urease and hemolysins are critical for *P mirabilis*. The experiments demonstrated concentration-dependent inhibition of both urease and hemolysin production by EGCG (Fig. 2).

### 3.3. EGCG’s impact on P mirabilis biofilm formation

**Figure 3.**
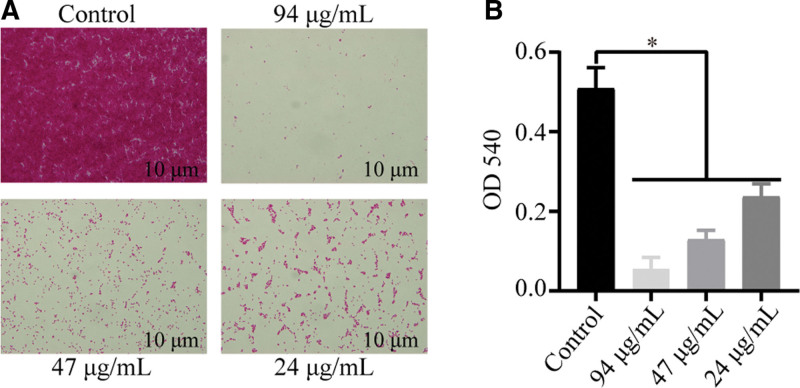
Inhibition of biofilm formation by EGCG. (A) Microscopic images demonstrating the inhibitory effect of EGCG on biofilm formation. (B) Quantification of biofilm inhibition by EGCG, assessed via crystal violet staining assay. Data are mean ± standard deviation from 3 biological replicates. Asterisks denote statistically significant differences (*P < .05). EGCG = epigallocatechin gallate.

Experiments showed that EGCG inhibited *P mirabilis* biofilm formation in a concentration-dependent manner, with the most pronounced effect observed at the MIC. Optical microscopy revealed that biofilms treated with EGCG exhibited a looser structure than those in the control group (Fig. 3).

### 3.4. EGCG’s influence on P mirabilis crystal production

**Figure 4.**
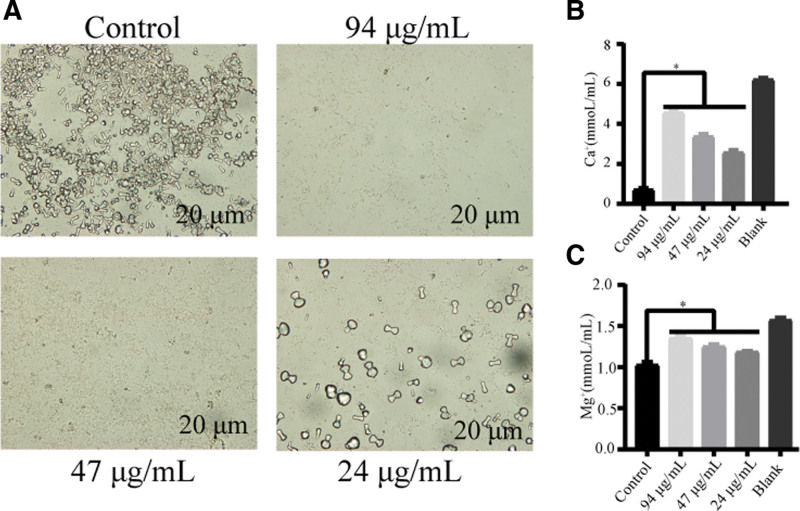
EGCG’s impact on crystal formation. (A) Microscopic images illustrating EGCG’s inhibitory effect on crystal formation. (B, C) Concentrations of calcium and magnesium ions in culture media after EGCG treatment. Data from 3 biological replicates are shown as mean ± standard deviation. Significant differences are marked with asterisks (*P < .05). EGCG = epigallocatechin gallate.

The effect of EGCG on *P mirabilis* crystal formation was investigated, considering the crucial role of crystal formation in CAUTIs. The culture medium from the EGCG-treated groups displayed a notably higher content of calcium and magnesium ions than the control group, whereas the crystal content observed on the slides was reduced. These findings indicate a significant inhibition of crystal formation by EGCG in *P mirabilis* (Fig. 4).

### 3.5. EGCG’s impact on P mirabilis swarming motility

**Figure 5.**
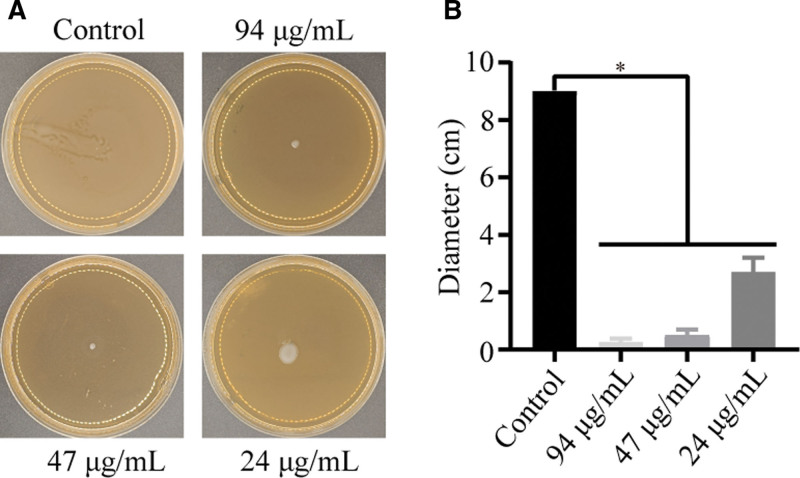
EGCG reduces swarming motility in *Proteus mirabilis*. (A) Images showcasing the inhibitory effect of EGCG on swarming motility. (B) Analysis of the migration distance of *P mirabilis* across different EGCG concentrations. Data are mean ± standard deviation from 3 biological replicates, with asterisks indicating significant differences (*P < .05). EGCG = epigallocatechin gallate.

Swarming motility is a key pathogenic trait in *P mirabilis*. The analysis demonstrated a negative correlation between EGCG concentration and swarming diameter, with LB agar images providing evidence of the effect of EGCG on the swarming motility of *P mirabilis* (Fig. 5).

### 3.6. EGCG’s effect on P mirabilis gene expression

**Figure 6.**
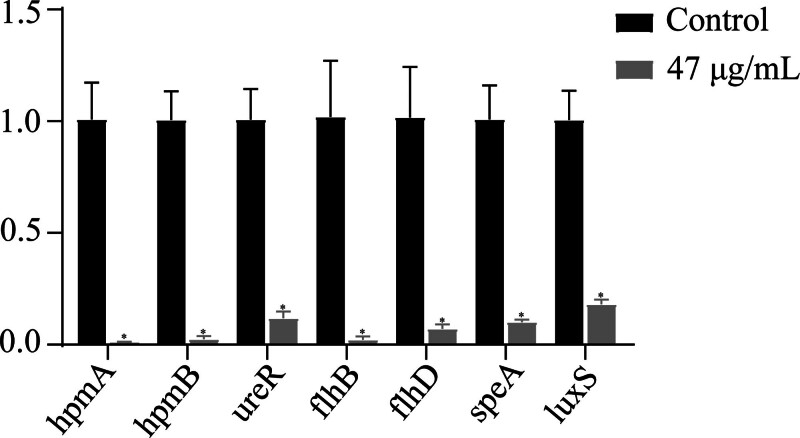
EGCG effects on gene expression. Graphic representation of gene expression changes induced by EGCG, with data expressed as mean ± standard deviation. Significant differences between treated and control samples are indicated with asterisks (*P < .05). EGCG = epigallocatechin gallate.

qRT-PCR analysis was conducted to assess changes in the expression of key virulence genes in *P mirabilis* following treatment with 47 μg/mL EGCG (Fig. 6). The findings demonstrated a downregulation of genes implicated in the synthesis of urease and hemolysin (*ureR*, *hpmA*, and *hpmB*). Furthermore, the genes responsible for swarming motility (*flhB*, *flhD*, and *speA*) showed decreased expression. Additionally, a reduction in expression was observed in genes associated with quorum sensing (QS), specifically *luxS*, indicating a broad inhibitory effect of EGCG on virulence gene expression in *P mirabilis*.

## 4. Discussion

The mechanisms underlying *P mirabilis*-induced ureteral-related CAUTIs are notably complex. Traditional approaches, including conventional antibiotic treatment and catheter replacement, often fail to manage these infections effectively. Focusing on the virulence factors of a pathogen has emerged as a promising strategy to mitigate its pathogenicity.^[5]^ This study revealed a significant concentration-dependent inhibitory effect of EGCG on the growth and virulence factor production of *P mirabilis*. Importantly, the gene expression analysis was conducted using 47 μg/mL EGCG, a concentration below the MIC. By adopting this sub-MIC level, we demonstrated that EGCG attenuates virulence gene expression without imposing strong bactericidal pressure. This suggests that the inhibitory effects are attributable to interference with bacterial regulatory pathways rather than growth inhibition alone, underscoring EGCG’s potential as an anti-virulence agent in CAUTIs.

*P mirabilis* is known for its propensity to form biofilms on urinary catheter surfaces, which complicates CAUTI treatment.^[27,28]^ Although traditional antibiotics, such as ciprofloxacin, at sub-MIC levels have been effective in reducing biofilm formation by up to 93%, as observed by Guttenplan et al, increasing antibiotic resistance poses a significant challenge.^[28]^ This study indicates that EGCG can effectively inhibit *P mirabilis* biofilm formation. Microscopic examination revealed a looser biofilm structure on slides treated with EGCG, consistent with the observations of Guttenplan et al,^[29]^ suggesting that EGCG is a viable alternative to antibiotics. Moreover, the swarming motility of *P mirabilis* facilitates retrograde infection of solid surfaces. The antibiofilm activity of yellow tea polyphenols, including EGCG, has been attributed to their ability to inhibit swarming motility.^[30]^ Research by O’May C demonstrated that EGCG hindered the swarming motility of *Pseudomonas aeruginosa*, thereby suppressing biofilm formation and resistance to tobramycin.^[31]^ The present study corroborates these findings, showing a significant reduction in swarming motility by EGCG, suggesting that its antibiofilm efficacy may stem from its ability to impede community differentiation.

Urease and hemolysin, which are critical for the pathogenicity of *P mirabilis*, facilitate urea hydrolysis, leading to the formation of struvite and hydroxyapatite crystals. These processes can result in kidney stones and tube blockages.^[32,33]^ Inhibiting of urease production, thereby preventing an increase in pH, is a crucial strategy for preventing the formation of crystalline biofilms. Ranjbar-Omid et al demonstrated that sub-MIC levels of allicin could reduce crystalline biofilms by 35%, contributing to the ability of allicin to penetrate bacterial membranes and to inhibit intracellular urease.^[13]^ This study observed a substantial inhibition of urease and hemolysin formation with EGCG treatment, further supported by microscopic examinations and calcium-magnesium ion concentration measurements in the culture medium, indicating EGCG’s role of EGCG in the suppression of crystal formation. Such treatment may delay urethral obstruction in *P mirabilis* infections.

To explore the molecular mechanisms underlying EGCG’s inhibition of virulence factors in *P mirabilis*, qRT-PCR analysis was performed to examine the expression of genes encoding these virulence factors. Treatment with 47 μg/mL EGCG significantly downregulated the expression of virulence-associated genes in *P mirabilis*, consistent with the findings of Mirzaei et al.^[34]^ Swarming motility of *P mirabilis* on solid surfaces is predominantly mediated by flagella. *flhD* and *flhB* regulate flagellar motility, where *flhD* binds to the *flhB* promoter region to regulate flagellin monomer expression and energy acquisition for flagellar rotation.^[7,35]^ The qRT-PCR analysis in this study revealed a significant downregulation in the expression levels of *flhD* and *flhB* following EGCG treatment, consistent with the results of the in vitro swarming motility assay. Additionally, *speA* (encoding arginine decarboxylase) is involved in the swimming and swarming motility of *P mirabilis*, aiding the use of intracellular protons to drive flagellar rotation.^[4]^ Gene expression analysis revealed a significant decrease in *speA* expression following EGCG treatment.

This study further investigated the effect of EGCG on urease and hemolysin expression, revealing significant inhibition attributed to the downregulation of *ureR*, *hpmA*, and *hpmB*. Urease activity, regulated by the transcriptional regulator *UreR* and the subsequent activation of the urease operon (ure gene cluster) are critical for the pathogenicity of *P mirabilis*.^[36,37]^ The findings from this study suggest significant inhibition of urease expression, which is attributed to the downregulation of *ureR* by EGCG. Likewise, *hpmA* and *hpmB*, which are essential for hemolysin production, were downregulated after EGCG treatment. This regulation likely contributes to tissue damage mitigation, typically caused by *HpmA* hemolysin, with *HpmB* playing a role in its activation and transport.^[8]^ These results are consistent with the observed in vitro inhibition of urease and hemolysin production by EGCG.

The inhibition of QS and initial cell attachment, which are critical for biofilm development and cell adhesion, were also examined. Previous research, including that by Šimunović K et al^[38]^ has shown that natural extracts such as basil, nettle, and rosemary can modulate the QS system and reduce motility and adhesion in pathogens such as *Clostridium difficile*. The findings of this study revealed that EGCG treatment leads to the downregulation of genes involved in QS, suggesting a mechanism by which EGCG suppresses quorum sensing and consequently diminishes biofilm formation.^[37,39,40]^ This mechanism corresponds with the reduction in biofilm formation we observed, underscoring that EGCG’s antibiofilm activity is likely mediated through its inhibition of QS gene expression.

The findings of this study significantly enhance our understanding of the inhibitory effects of EGCG on growth and virulence factors of *P mirabilis*. These insights pave the way for novel approaches to prevent and treat CAUTIs caused by *P mirabilis*. Future investigations will explore the in vivo antimicrobial efficacy of EGCG using experimental animal models and assess the toxicity of its active components, aiming to comprehensively delineate its therapeutic potential.

## Acknowledgments

The authors are grateful to the Ningde Municipal Hospital of Ningde Normal University, Ningde, China, for the financial support granted to cover the publication fee for this research article.

## Author contributions

**Conceptualization:** Guiju Fang.

**Data curation:** Yuying Qi, Qin Zeng.

**Investigation:** Qin Zeng.

**Resources:** Gang Yan.

**Software:** Zhichun Xue, Huiling Chen.

**Writing – original draft:** Zhichun Xue, Huiling Chen.

**Writing – review & editing:** Guiju Fang.
